# Active Films Based on Orange Peel Pomace Soluble Dietary Fiber and Sodium Alginate Reinforced by Dual-Functional Cellulose Nanocrystal/Zinc Oxide Hybrids for Antibacterial Food Packaging

**DOI:** 10.3390/foods15213890

**Published:** 2026-10-30

**Authors:** Lili Chen, Yuqi Hu, Yincai Wu, Xinjia Jiang

**Affiliations:** 1 Arts Institute, Hengyang Normal University, Hengyang 421002, China; 2 Xiamen Institute of Rare Earth Materials, Chinese Academy of Sciences, Xiamen 361021, China; 3 School of Packaging Engineering, Hunan University of Technology, Zhuzhou 412007, China

**Keywords:** orange peel pomace, soluble dietary fiber, cellulose nanocrystal/ZnO hybrid, blueberry preservation

## Abstract

In this study, dual-functional cellulose nanocrystal/zinc oxide hybrids (ONCC@ZnONP) were constructed from navel orange peel pomace. The incorporation of these hybrids systematically improved the overall properties of OSDF-based antibacterial active packaging films. A series of antibacterial active packaging films (O/CZ) were successfully prepared using OSDF as the matrix and ONCC@ZnONP as the active filler. The effects of different ONCC@ZnONP loadings on the microstructure, physical properties, and antibacterial properties of the films were systematically analyzed. The results showed that ONCC@ZnONP was uniformly dispersed within the OSDF matrix, exhibiting favorable interfacial compatibility. The incorporation of ONCC@ZnONP significantly enhanced the tensile strength, UV-shielding property, and antibacterial activity of the O/CZ films. In the blueberry preservation application, the decay rate of the O/CZ films decreased with increasing ZnO loading. Based on the comprehensive balance of antibacterial preservation and physical quality maintenance, the O/CZ8 film (8 wt% ONCC@ZnONP) showed the optimal preservation performance. Specifically, it showed a weight loss of 9.67%, maintained a firmness of 9.07 N, and achieved a decay rate reduction of 78.74% compared to the OSDF/SA film. These results showed that the developed antibacterial active packaging films hold great potential for food packaging and preservation applications.

## 1. Introduction

The excessive use of traditional non-biodegradable plastics in packaging led to severe environmental pollution and health risks. Consequently, natural biopolymer-based food packaging films became a research focus in both academia and industry [1,2,3,4,5]. These films utilized natural macromolecules (e.g., polysaccharides, proteins, and lipids) as the matrix and were supplemented with food-grade plasticizers. They were fabricated through processes such as blending, heating, and extrusion, during which the components formed structurally dense films via intermolecular interactions or crosslinking reactions [6]. However, active packaging films based on biopolymers showed several drawbacks, including poor mechanical properties, inferior barrier performance, limited functionality, and high susceptibility to microbial contamination [7,8,9,10,11]. Therefore, to impart active functionality, active substances capable of inhibiting microbial growth on food surfaces were generally incorporated into food packaging films. Consequently, zinc oxide nanoparticles (ZnONPs) [12,13,14] or cellulose fiber/ZnONP hybrids [15,16,17] were frequently introduced into polymer matrices such as sodium alginate. This endowed the films with antibacterial activity while simultaneously improving their mechanical properties and UV-shielding performance.

Navel orange (*Citrus sinensis*) belonged to the sweet orange species of the genus Citrus. It possessed multiple health-promoting effects, such as lipid-lowering, skin-beautifying, and anticancer properties, and was acclaimed as the “King of Citrus Fruits” [18]. Market statistics indicated that approximately 55% of navel oranges were sold as fresh fruit. Meanwhile, 35% to 40% were directed into juice and canning processing, which generated substantial quantities of peel pomace [19]. These by-products were mostly landfilled directly or processed into low-value animal feed. This resulted in both resource waste and environmental pressure [20,21]. In fact, navel orange peel pomace served as a rich source of dietary fiber. Specifically, soluble dietary fiber (OSDF) accounted for up to 19.56%, and insoluble dietary fiber reached 76.98% [22]. Although OSDF possessed good hydration and film-forming properties [23], a single OSDF matrix had low crosslinking density. This restricted its structural stability and mechanical strength. To address this, sodium alginate (SA) was introduced. Its G-blocks coordinated with the added Ca^2+^ to form a classic “egg-box” ionic crosslinking network [24]. This network enhanced the structural density of the matrix and provided a foundation for filler dispersion and stress transfer. Based on this, cellulose nanocrystals (ONCC) extracted from insoluble dietary fiber served as a biotemplate. ZnO was anchored in situ to construct the dual-functional ONCC@ZnONP filler. This study focused on the structure–property relationship of this filler. Since the Ca^2+^-crosslinked SA network served as a fixed base matrix, its crosslinking degree was not evaluated as an independent variable. This strategy provided a novel pathway for the green and high-value utilization of navel orange processing waste. It also offered new insights for material design and preparation in food packaging films.

Due to their high aspect ratio, high crystallinity, and abundant surface hydroxyl groups, cellulose nanocrystals (NCC) emerged as ideal organic reinforcing phases for food packaging films. They significantly enhanced the mechanical strength and gas/water vapor barrier properties of the matrix through a synergistic effect of physical filling and interfacial hydrogen bonding/van der Waals forces [25,26,27,28]. However, NCC inherently lacked antibacterial activity and had limited UV-shielding capability. These limitations restricted their further application in functional packaging. Zinc oxide nanoparticles (ZnONP) were considered preferred inorganic additives for antibacterial food packaging due to their low cost, safety at compliant dosages, and ease of scalable preparation. They also possessed high thermal stability, broad-spectrum antibacterial properties, and strong UV absorption capabilities [29,30,31]. However, ZnONP were prone to self-agglomeration, which led to a decline in their activity [32]. Using cellulose nanocrystals as templates, anchoring ZnONP onto the cellulose backbone via in situ growth or electrostatic self-assembly suppressed agglomeration and achieved organic–inorganic synergistic reinforcement [33,34]. Although Chen et al. [35] reported OSDF/SA/ONCC films based on navel orange peel soluble dietary fiber/sodium alginate, the ONCC primarily acted as a physical reinforcer. They inherently lacked antibacterial and UV-shielding capabilities, making it difficult to actively inhibit food surface microorganisms, thus limiting their preservation effects on highly perishable fruits. Furthermore, existing CNC/ZnO hybrid studies mostly focused on matrices such as PVA, PLA, PHBV, gelatin, and soy protein isolate. In these systems, ZnO was often introduced via physical blending, which readily caused agglomeration. This resulted in poor filler–matrix interfacial compatibility and trade-offs among mechanical, optical, and barrier properties [36,37,38].

In this study, cellulose nanocrystals from navel orange peel pomace (ONCC) served as a biotemplate. Cellulose nanocrystal/zinc oxide nanoparticle hybrids (ONCC@ZnONP) were synthesized in situ via a low-temperature, one-step hydrothermal method. OSDF served as the film-forming matrix and SA as the co-film former. ONCC@ZnONP acted as an active filler with dual antibacterial and reinforcing functions. Based on this, the OSDF/SA/ONCC@ZnONP antibacterial active packaging films (O/CZ films) were successfully prepared. The effects of different ONCC@ZnONP loadings on the microstructure, physical properties, and antibacterial activity of the O/CZ films were systematically investigated. Their application in blueberry preservation was evaluated, and the filler loading–structure–property relationship was elucidated. This work provided a new pathway for the high-value utilization of navel orange processing by-products. It also validated the effectiveness of the O/CZ films in extending blueberry freshness and shelf life, offering new insights for the field of active food packaging.

## 2. Materials and Methods

### 2.1. Materials and Reagents

The navel orange peels were provided by Guoran Food Co., Ltd. (Ganzhou, China). The soluble dietary fiber (OSDF) and cellulose nanocrystals (ONCC) from navel orange peel pomace, as well as sterile deionized water, were all prepared in-house in our laboratory. Sodium alginate (SA, the viscosity was 350–550 cP) and glycerol (Gly, the relative molecular mass was 92.09, the content was ≥99.0%, and the chromaticity was ≤10) were purchased from Shanghai Aladdin Biochemical Technology Co., Ltd. (Shanghai, China). Anhydrous zinc chloride (ZnCl_2_, the purity was 98%) was obtained from Beijing Innochem Technology Co., Ltd. (Beijing, China). Absolute (anhydrous) ethanol was obtained from Hunan Huihong Reagent Co., Ltd. (Changsha, China); all the foregoing reagents were of analytical grade. Food-grade calcium chloride (CaCl_2_, the molecular weight was 110.98, and the relative density was 2.15) was purchased from Henan Wanbang Chemical Technology Co., Ltd. (Zhengzhou, China). Yeast extract, peptone, agar powder (biochemical grade), as well as sodium hydroxide and sodium chloride (analytical grade), were all purchased from Sinopharm Chemical Reagent Co., Ltd. (Shanghai, China). The bacterial strains *Staphylococcus aureus* (ATCC 25923) and *Escherichia coli* (ATCC 25922) were provided by the Microbiology Laboratory, College of Life Science and Chemistry, Hunan University of Technology (Zhuzhou, China).

### 2.2. Preparation of ONCC and ONCC@ZnONP

First, soluble dietary fiber (OSDF, D_50_ = 156.78 μm, SSA = 7.85 m^2^/g) and insoluble dietary fiber (OIDF, D_50_ = 244.47 μm, SSA = 1.35 m^2^/g) were extracted from navel orange peel pomace by an ultrasound-assisted complex enzymatic method, in which food-grade lipase, papain, medium-temperature amylase, and cellulase were used [22]. Second, ONCC was prepared from OIDF using an organic acid (oxalic acid). Briefly, a 30 mL solution of 60 wt% oxalic acid was prepared and magnetically stirred until completely dissolved, after which a certain amount of OIDF was added and reacted for 180 min at 115 °C. After the reaction, deionized water at 80◦C was added to terminate the reaction. The solution was then repeatedly washed by vacuum filtration and centrifugation until the pH became neutral. To obtain a turbid solution and precipitated cellulose, deionized water was added to the centrifuged cellulose suspension, and the suspension was ultrasonicated for 15 min (600 W, 20 kHz; 5 s on, 5 s off); this step was repeated twice. Finally, the turbid suspension was centrifuged once more to remove the residue and obtain a clear ONCC suspension (0.14% solid content). The ONCC suspension was kept at 4 °C for later use.

The preparation method for ONCC@ZnONP was based on the protocol reported by Abdalkarim et al. [39] with further optimization. In this study, ONCC@ZnONP was synthesized in situ via a mild hydrothermal reaction using ONCC as a biological template. First, 5 mmol of ZnCl_2_ (0.6815 g) and 15 mmol of NaOH (0.6 g) were dissolved in 6 mL of deionized water. The solution was vigorously stirred at 800 rpm for 15 min at room temperature to obtain a homogeneous precursor solution. Subsequently, 29 mL of an ONCC suspension was pre-treated by probe ultrasonication (600 W, 5 min, 5 s on/off cycle). This suspension was slowly added dropwise into the precursor solution. The mixture was continuously stirred at 800 rpm for another 30 min to ensure thorough complexation. The resulting mixed system was then transferred to a constant temperature water bath and allowed to react statically at 80 °C for 24 h. After the reaction, the product was washed sequentially with deionized water and anhydrous ethanol 3 to 5 times (centrifugation at 8000 rpm with a centrifugation radius of 9.97 cm, 10 °C for 10 min). The obtained precipitate was dried at 60 °C for 12 h to yield the crude ONCC@ZnONP product. Finally, it was further dried at 120 °C for 1 h to completely dehydrate the residual zinc hydroxide into zinc oxide. The purified and dried ONCC@ZnONP hybrid was thus obtained. The theoretical mass ratio of ZnO to ONCC in ONCC@ZnONP was approximately 10:1 (about 90.9 wt% ZnO and 9.1 wt% ONCC).

### 2.3. Preparation of the Antibacterial Active Packaging Films

The OSDF/SA and OSDF/SA/ONCC packaging films were prepared following the method of Chen et al. [35] with minor modifications. For the OSDF/SA packaging films, the formulation was as follows: the total dry weight of the film was fixed with a solid-to-liquid ratio of 1:25 (*w*/*v*). Specifically, 0.3 g of the OSDF and SA mixture (containing 0.17 g of OSDF) was used, and glycerol (Gly) was added at a concentration of 25% (*w*/*w*). The OSDF/SA/ONCC packaging films were prepared by incorporating a mass fraction of 1% ONCC suspension into the OSDF/SA composite film-forming solution.

The O/CZ films were developed through a multi-step procedure. First, an OSDF/SA composite solution was prepared as described for the blank OSDF/SA film. Subsequently, ONCC@ZnONP powders were weighed at mass fractions of 2, 4, 6, 8, and 10 wt% based on dry weight, respectively. These loadings corresponded to theoretical ZnO contents of approximately 1.82–9.09 wt% and ONCC contents of 0.18–0.91 wt%. These powders were added into 10 mL of deionized water and fully dispersed by probe ultrasonication (600 W, 5 min; 5 s on, 5 s off cycle). The dispersion was subsequently poured into the OSDF/SA composite solution. This mixture was sequentially subjected to high-speed shearing (800 rpm, 10 min), probe ultrasonication (5 min), and magnetic stirring (30 min) to ensure uniform dispersion of the hybrid. After adding 0.1 g of glycerol as a plasticizer with magnetic stirring for 10 min, 10 mL of CaCl_2_ solution (0.1%, *w*/*v*) was added dropwise at 1 mL·min^−1^ to crosslink the SA, followed by 20 min of stirring. The resulting film-forming solution was degassed by ultrasonication for 30 min, cast into glass Petri dishes (60 mm diameter), and dried at 55 °C for 24 h. Finally, the films were conditioned at 25 ± 1 °C and 60% relative humidity for at least 24 h prior to characterization.

**Figure 1:**
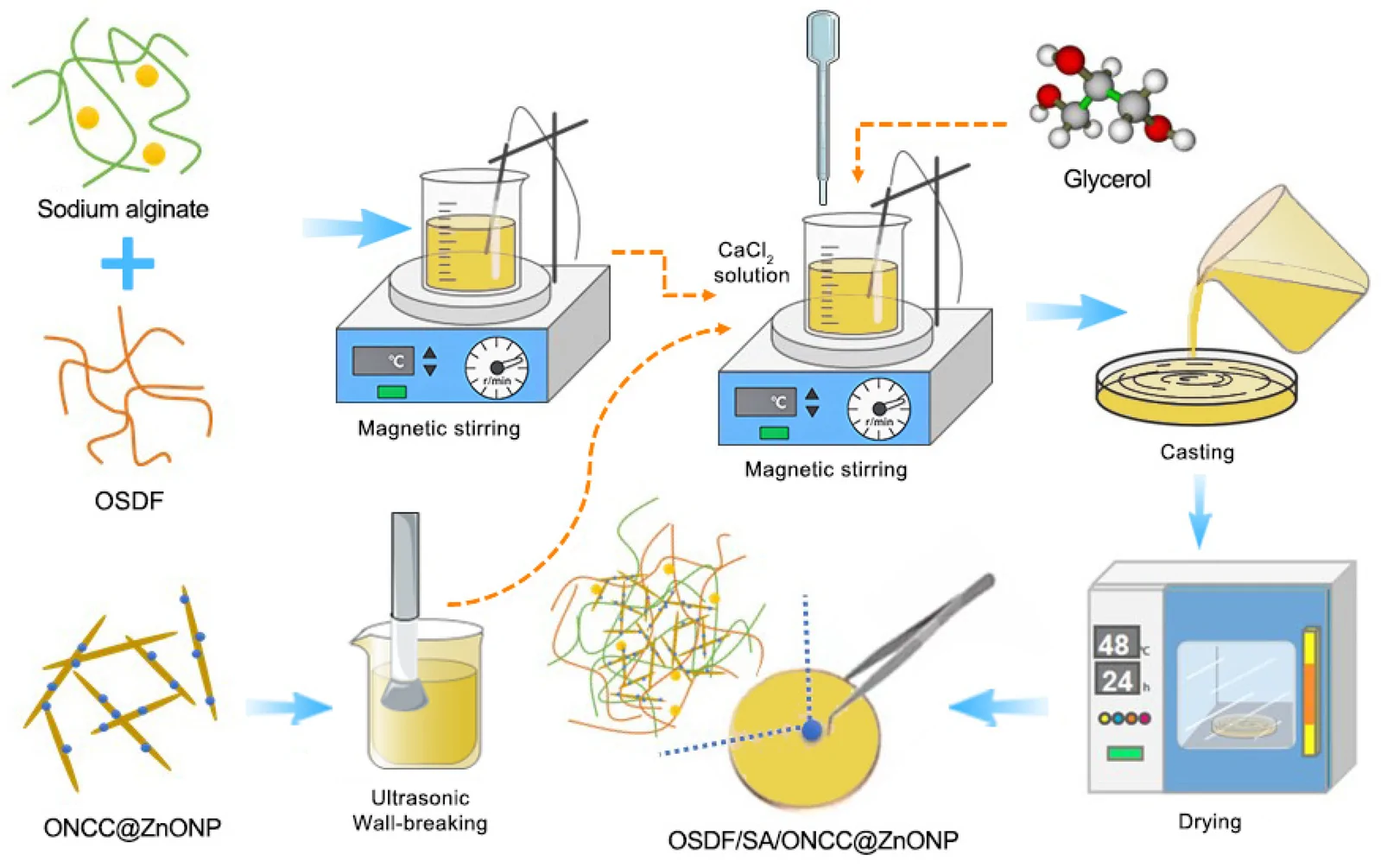
The flow scheme of the whole process for preparing O/CZ films.

The antibacterial active packaging films, with ONCC@ZnONP contents of 2, 4, 6, 8, and 10 wt%, were designated as O/CZ2, O/CZ4, O/CZ6, O/CZ8, and O/CZ10 film, respectively. A schematic of the complete preparation process was shown in Figure 1.

### 2.4. Test Characterization

#### 2.4.1. Microstructural Morphology and Elemental Analysis

Field-emission scanning electron microscopy (FE-SEM, Apreo S, Waltham, MA, USA) equipped with an energy-dispersive X-ray spectroscopy (EDS) system was employed to observe the morphology and elemental distribution of ONCC@ZnONP. The surface and cross-sectional morphologies of the O/CZ films were examined using the same FE-SEM instrument. Samples were mounted on conductive tape, sputter-coated with gold for 90 s, and imaged at an accelerating voltage of 5 kV and a working distance of 5 mm.

#### 2.4.2. Chemical Structure

The O/CZ films were characterized by Fourier transform infrared spectroscopy (FTIR, Nicolet 380, Madison, WI, USA) in the wavenumber range of 4000–400 cm^−1^ at a resolution of 0.5 cm^−1^ with 32 scans.

#### 2.4.3. Crystalline Structure

X-ray diffraction (XRD) analysis (Akishima, Tokyo, Japan) was performed to investigate the crystalline properties of the samples, using CuKα radiation (λ = 0.15406 nm) with a Ni filter. Spectra were collected in the 2θ range of 5–50° at a scanning speed of 2° min^−1^ and a step size of 0.02°. The crystallinity index (CrI) was calculate using the Segal empirical formula:

CrI = (I_002_ − I_am_)/I_002_ × 100% (1)

where I_002_ represented the intensity of the diffraction peak at 22.6° and I_am_ represented the diffraction intensity of the peak in the amorphous region at 18°.

#### 2.4.4. Thermal Properties

The thermal stability of the samples was determined using a thermogravimetric analyzer (TGA, Q50, New Castle, DE, USA). The samples were heated from 35 °C to 600 °C at a heating rate of 10 °C/min under a nitrogen atmosphere, and the weight change was recorded as a function of heating temperature. The thermal behavior of the samples was characterized using a differential scanning calorimeter (DSC, Q20, New Castle, DE, USA). Samples of 5–7 mg were sealed in aluminum pans and heated from 30 °C to 180 °C at a rate of 5 °C/min under a nitrogen atmosphere (the flow rate was 50 mL/min), held at 180 °C for 1 min, and then cooled to 30 °C at 10 °C/min. The heat flow signal was recorded throughout the entire process. All DSC curves and thermal parameters presented in this study, including the glass transition temperature (T_g_), were obtained from the first heating scan. The T_g_ was determined according to the equal-area method specified in ISO 11357-2:2020 [40].

#### 2.4.5. Mechanical Properties

Tensile properties of the O/CZ films were tested using a universal testing machine (ETM 502B-EX, Shenzhen, China). The films were cut into strip specimens of 6 mm × 35 mm, and their thickness was measured at five points using a micrometer to obtain an average value. Measurements were carried out at a crosshead speed of 2 mm·min^−1^ with an initial gauge length of 20 mm. Five replicates per group were tested, and the results were presented as mean ± standard deviation.

#### 2.4.6. Optical Properties

The optical properties of the O/CZ films were evaluated by measuring light transmittance using a UV-Vis spectrophotometer (Lambda 950, Waltham, MA, USA) in the wavelength range of 200–800 nm, with a 1 cm optical path and air as the reference.

#### 2.4.7. Antibacterial Performance Testing

Gram-positive *Staphylococcus aureus* (*S. aureus*) and Gram-negative *Escherichia coli* (*E. coli*) were selected as model strains, and a bacterial suspension with a uniform fixed concentration of 1 × 10^6^ CFU/mL was used for the antibacterial experiments. The OSDF/SA packaging films were used as controls groups. Prior to testing, all film samples were UV-sterilized on both sides for 20 min.

Inhibition zone test (qualitative): Referring to the method of Azizi et al. [41], the film samples were cut into discs with a diameter of 6 mm. LB agar plates were inoculated with 100 μL of bacterial suspension, and the film discs were placed onto the agar surface. Following incubation at 37 °C for 24 h, the diameters of the resulting zones of inhibition (ZOIs) were measured using a vernier caliper.

Plate counting test (quantitative): According to the method of Feng et al. [42], 0.05 g of sample was accurately weighed into a sterile centrifuge tube and mixed with 5 mL of diluted bacterial suspension. The mixture was incubated with shaking at 180 rpm and 37 °C for 24 h. After incubation, the mixture was serially diluted with sterile water, plated onto LB agar, and incubated at 37 °C for 24 h before colony counting.

### 2.5. Application of O/CZ Films in Blueberries Preservation

Fresh blueberries with uniform size, color, and maturity were selected and verified to be free from insect damage and mechanical injury. After wiping the surfaces with sterile paper, they were randomly divided into seven treatment groups. Each group contained 7 blueberries, with 3 independent packaging replicates (*n* = 3). Different films (OSDF/SA, OSDF/SA/ONCC, O/CZ2, O/CZ4, O/CZ6, O/CZ8, and O/CZ10) were used as sealing films. They were placed over and sealed onto the surfaces of Petri dishes containing the blueberries. All samples were stored for 16 days under normal indoor scattered light at 20 ± 1 °C and 60% relative humidity. The indicators were measured every 4 days.

#### 2.5.1. Appearance Changes

The appearance changes of the blueberries was recorded using a digital camera (Nikon Z8, Nikon Corporation, Tokyo, Japan) at 0, 4, 8, 12, and 16 days of storage.

#### 2.5.2. Decay Rate

Fruit decay was determined by the presence of visible lesions and softening of injured areas. The decay rate (DR) was calculated according to the following equation:

DR (%) = (A/B) × 100%(2)

where A represents the number of rotten fruits, and B denotes the total number of fruits.

#### 2.5.3. Weight Loss

The weight loss rate was measured by the gravimetric method and calculated according to the following equation:

WL (%) = [(W_0_ − W_i_)/W_0_] × 100% (3)

where W_0_ represented the initial mass (g) on day 0, and W_i_ denoted the mass (g) measured after i days of storage.

#### 2.5.4. Firmness

The firmness of blueberry samples was measured using a fruit firmness tester (GY-4, Hangzhou Lvbo Instrument Co., Ltd., Hangzhou, China) equipped with an 11.1 mm diameter probe. The test speed was set at 1 mm·s^−1^, and the results were recorded in Newtons (N).

### 2.6. Statistical Analysis

Except for the blueberry preservation indicators, the material characterization data of other single variables were analyzed using one-way analysis of variance (One-way ANOVA). The blueberry preservation indicators (decay rate, weight loss, and firmness) were analyzed using two-way analysis of variance (Two-way ANOVA). This was performed to evaluate the effects of film treatment, storage time, and their interaction on the indicators. Duncan’s multiple range test was used for post-hoc multiple comparisons of all data. Statistical analyses were performed using SPSS software (IBM SPSS Statistic 26, Armonk, NY, USA). Differences were considered statistically significant at *p* < 0.05.

## 3. Results and Discussion

### 3.1. Microstructure and Elemental Composition of ONCC@ZnONP

**Figure 2:**
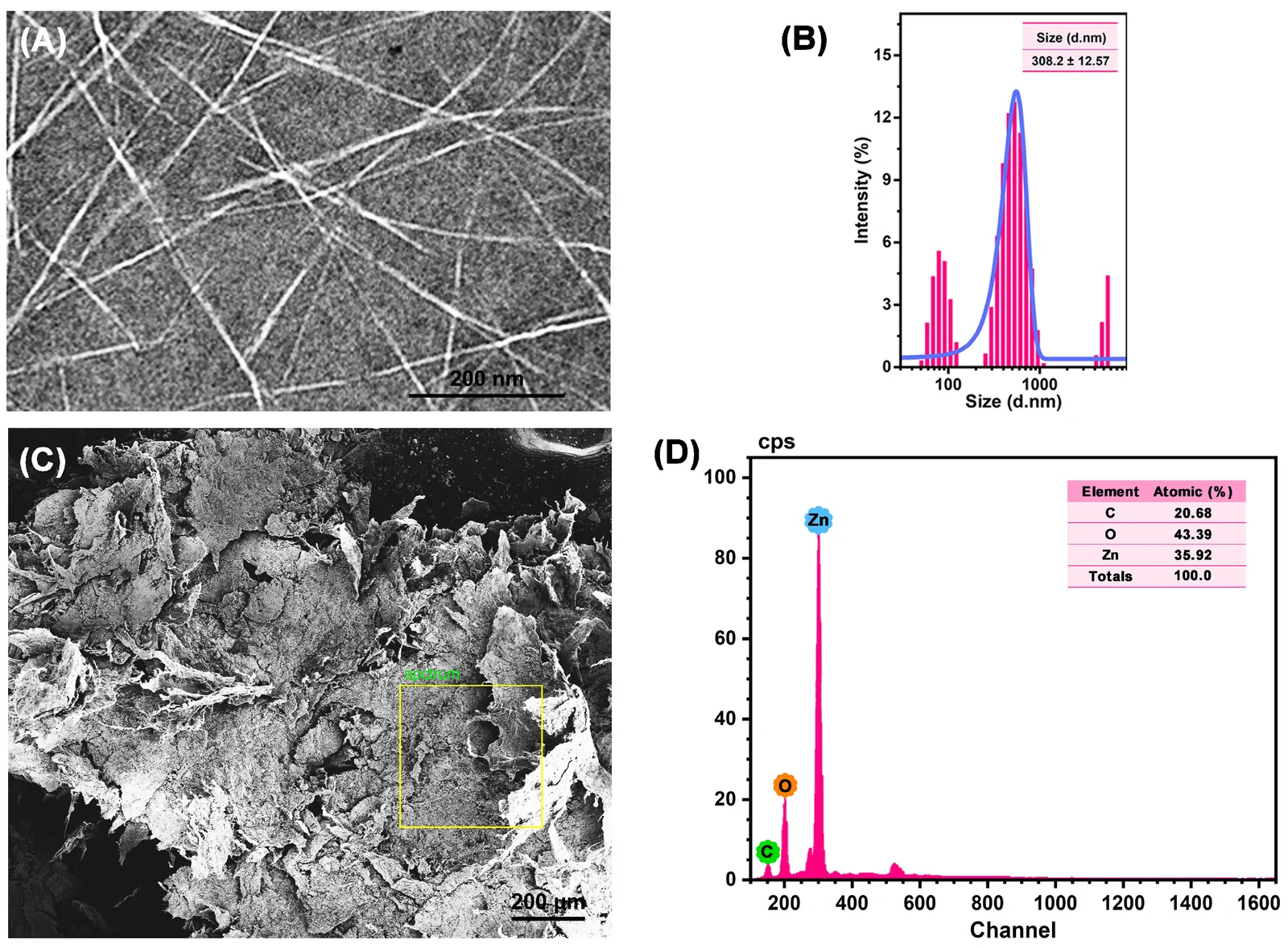
(**A**) TEM image and (**B**) particle size of ONCC, (**C**) SEM, and (**D**) EDS spectra of ONCC@ZnONP.

As shown in Figure 2A, ONCC showed a needle-like structure with lengths ranging from 100 to 1000 nm and a diameter of approximately 4 nm. The particle size distribution of ONCC was showed in Figure 2B. The particles were mainly concentrated within the range of 100–1000 nm, with an average particle size of 308.20 nm, which was consistent with the statistical results obtained from the TEM image in Figure 2A. Figure 2C,D showed the morphological and elemental characterization results of ONCC@ZnONP, respectively. As shown in Figure 2C, ONCC@ZnONP showed a typical flower-like hierarchical structure self-assembled from numerous nanosheets. This morphology was significantly different from the needle-like shape of raw ONCC, indicating the formation of a new micro-nano self-assembled structure after ZnO deposition. The EDS spectrum in Figure 2D showed distinct characteristic peaks of Zn, C, and O. The atomic percentages (C: 20.68%, O: 43.39%, Zn: 35.92%) were consistent with the expected chemical composition of ZnO loaded onto the cellulose matrix. This confirmed the presence of ZnO and cellulose components in the hybrid, indicating their successful combination. ZnO and ONCC might be combined through electrostatic interactions [43]. A similar phenomenon was also verified in the CNC-ZnO nanohybrid system reported by Abdalkarim et al. [44].

### 3.2. SEM Analysis of O/CZ Films

**Figure 3:**
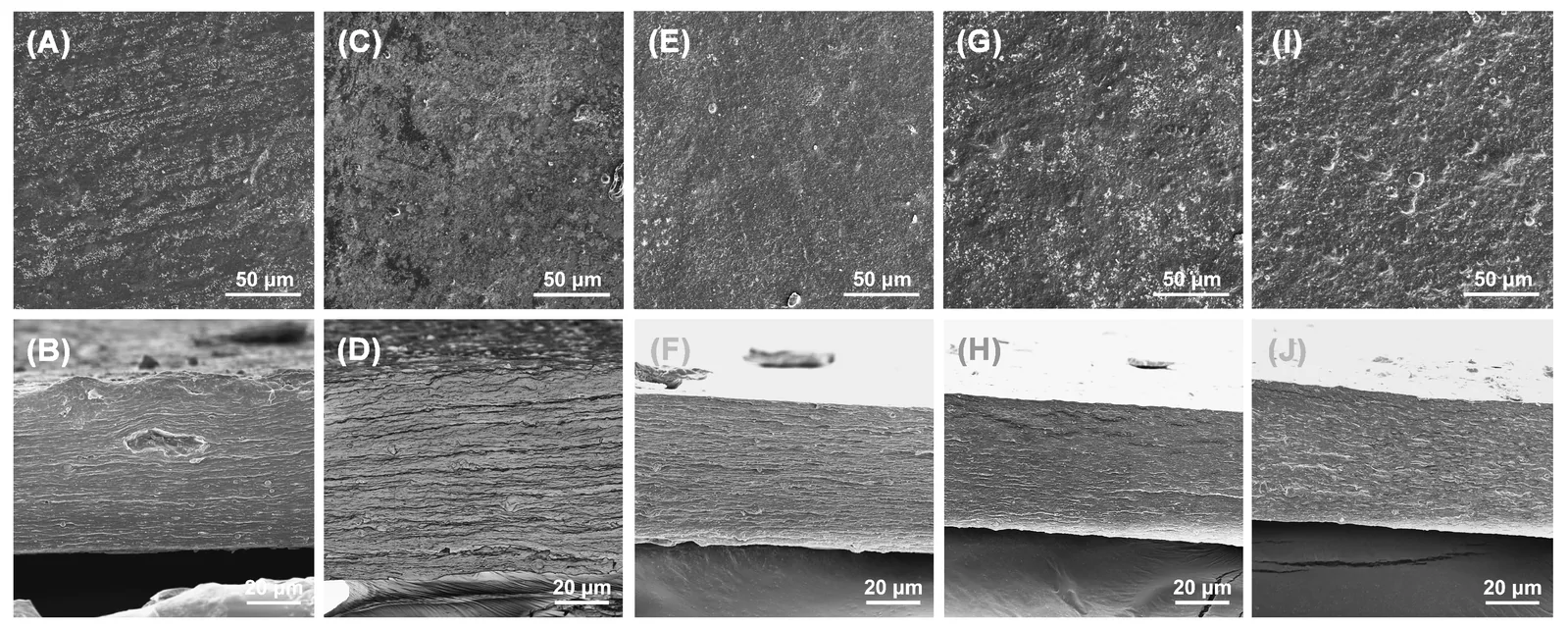
Surface and cross-section SEM images of (**A**,**B**) OSDF/SA film, (**C**,**D**) OSDF/SA/ONCC film, (**E**,**F**) O/CZ4 film, (**G**,**H**) O/CZ8 film, and (**I**,**J**) O/CZ10 film.

The dispersion state and interfacial bonding of ONCC@ZnONP within the OSDF matrix were investigated by field-emission scanning electron microscopy (FE-SEM). As shown in Figure 3, the low-magnification surface morphology images revealed a uniform distribution of hybrid particles in the OSDF matrix at relatively low loadings (e.g., the O/CZ2 and O/CZ4 films). No obvious pores or debonding were observed at the matrix-particle interface. This indicated good interfacial compatibility between the two components. As the ONCC@ZnONP content increased to 10 wt% (O/CZ10 film), the particle density on the composite film surface increased significantly. Localized micron-scale agglomeration occurred, suggesting that excessive filler loading led to a decrease in dispersion uniformity.

Cross-sectional observation further confirmed that ONCC@ZnONP was evenly embedded within the OSDF matrix and formed a dense interface. This compact structure effectively reduced the pathways for crack initiation and propagation and alleviated stress concentration. Consequently, it provided a structural foundation for the enhanced mechanical properties of the composite. Similar interfacial reinforcement phenomena were also reported in PLA-based composite film systems by Yu et al. [45]. This uniform dispersion and compact interfacial structure were consistent with the hydrogen-bonding interactions revealed by FTIR and the enhanced mechanical properties. It indicated that favorable filler–matrix compatibility serves as the structural basis for the improved tensile property of the O/CZ films.

### 3.3. FTIR Analysis of O/CZ Films

**Figure 4:**
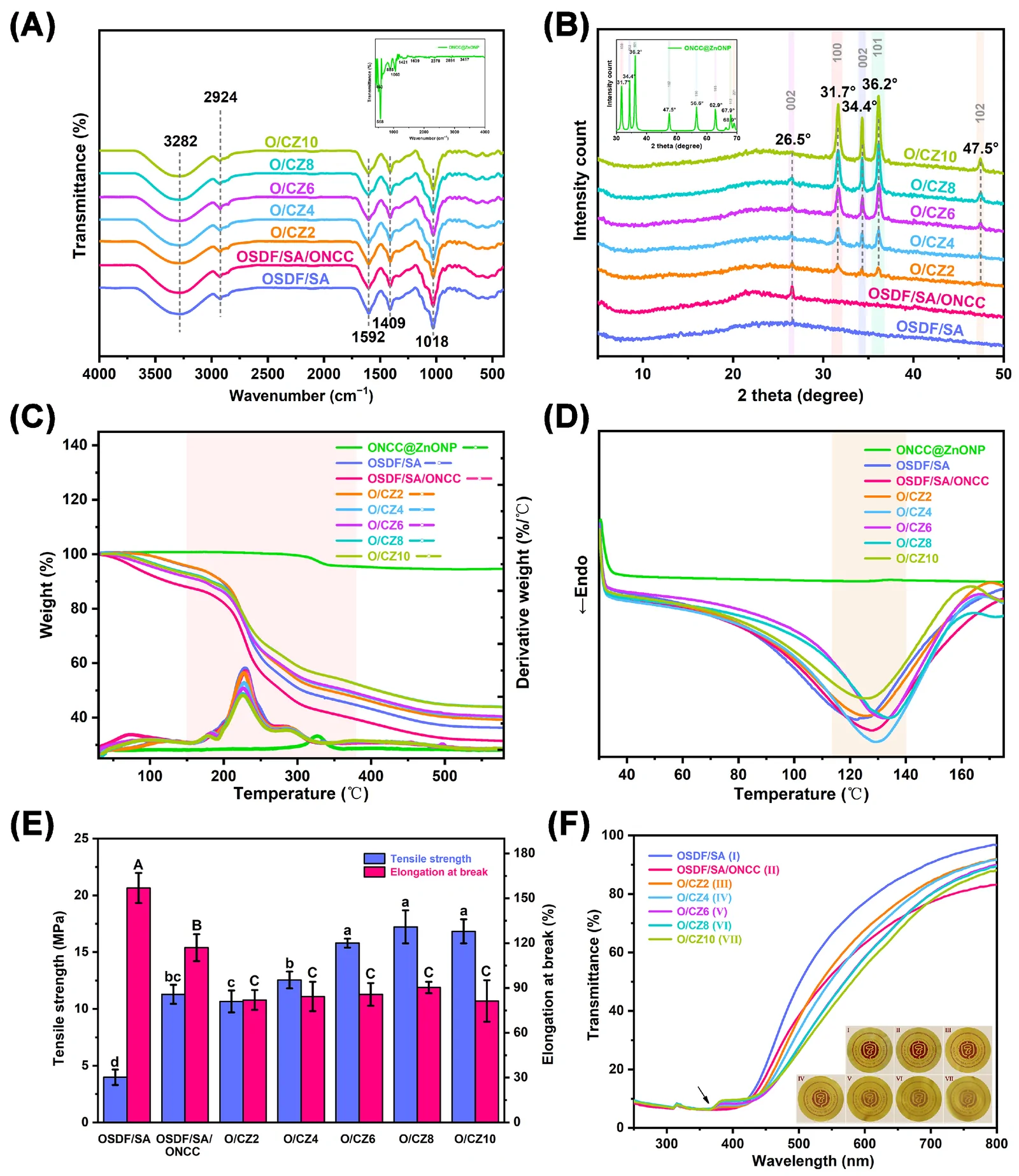
(**A**) FTIR spectra, (**B**) XRD, (**C**) TG, (**D**) DSC, (**E**) mechanical properties, and (**F**) optical properties of O/CZ. In (**C**), the shadowed area indicates the main thermal degradation stage of the polymer matrix. In (**D**), the shadowed area indicates the broad endothermic peak region. In (**E**), different letters on the bars indicate significant differences (*p* < 0.05). In (**F**), the arrow indicates the characteristic absorption band of ZnO at 365 nm.

The FTIR spectra of the O/CZ films were presented in Figure 4A. The characteristic spectrum of ONCC@ZnONP provided direct evidence for its chemical structure. The ONCC@ZnONP spectrum retained all characteristic absorptions of ONCC. A broad absorption peak at 3417 cm^−1^ was attributed to O–H stretching vibrations, while the bands at 1639 cm^−1^ and 1060 cm^−1^ corresponded to O–H bending vibrations and C–O–C cyclic ether stretching vibrations, respectively [46,47]. A weak peak at 2891 cm^−1^ originated from C–H stretching vibrations. This indicated the presence of alkyl structures in the ONCC molecular chains. Furthermore, characteristic absorptions of ZnO appeared at 450 cm^−1^ and 568 cm^−1^. The former corresponded to the longitudinal optical (LO) vibration mode of Zn–O bonds. This served as direct evidence of the strong interaction between Zn and O atoms in the ZnO lattice [48]. The latter was attributable to lattice phonon vibrations of Zn–O bonds, particularly those associated with bond angle deformation modes. A comparison of the spectra of the O/CZ films in Figure 4A showed that the positions of their main absorption peaks were essentially consistent with those of the OSDF/SA and OSDF/SA/ONCC films. No new absorption peaks were observed. The broadening of the hydroxyl peak in the FTIR spectrum suggested possible hydrogen-bonding interactions between ONCC@ZnONP and the OSDF matrix. This result was consistent with the increased apparent glass transition temperature (T_g_) in DSC and the improved tensile strength. It was speculated that interfacial non-covalent interactions restricted the segmental motion of OSDF chains. This contributed to the enhanced mechanical properties.

### 3.4. XRD Characterization of O/CZ Films

The XRD patterns of the O/CZ films and the ONCC@ZnONP hybrid were presented in Figure 4B. Sharp diffraction peaks were observed for ONCC@ZnONP at 2θ = 31.7°, 34.4°, and 36.2°. These peaks corresponded to the characteristic crystal planes of hexagonal wurtzite ZnO [49]. This confirmed the successful preparation and loading of ZnO. Both the OSDF/SA film and OSDF/SA/ONCC film showed a typical amorphous background without obvious crystalline peaks. This indicated that the interactions between ONCC and the OSDF matrix were likely non-covalent. With increasing ONCC@ZnONP content, the O/CZ films showed progressively more pronounced ZnO characteristic peaks at the same positions (2θ = 31.7°, 34.4°, and 36.2°). The peak shapes narrowed and the intensities enhanced. This directly reflected the effective loading of the hybrid within the films. Meanwhile, the matrix still maintained an amorphous background. This indicated that the non-covalent interactions between the filler and the matrix did not alter the amorphous structure of the matrix. Rahmadiawan et al. reported that the co-introduction of ginger nanocellulose and ZnO improved the crystallinity of PVA-based films [50]. However, in the present system, the enhanced crystalline signals in the XRD patterns of the O/CZ films were mainly attributed to the increased ZnO phase content. This was not due to an increase in matrix crystallinity. This result echoed the T_g_ variation observed in DSC. It further supported that the changes in thermal behavior mainly originated from the restriction of segmental motion by interfacial non-covalent interactions.

### 3.5. TG Analysis of O/CZ Films

TG and DTG analyses were conducted to investigate the thermal stability of the O/CZ films, and the results were presented in Figure 4C. All samples showed typical weight loss stages during heating. Evaporation of adsorbed water occurred below 120 °C. Thermal cracking of the matrix backbone occurred at 200–500 °C. Carbonization and inorganic residues occurred above 400 °C. ONCC@ZnONP maintained a high residual mass at 500 °C, indicating its excellent intrinsic thermal stability. ONCC@ZnONP was an inorganic–organic hybrid, and its incorporation increased the residual mass of the composite films. Therefore, decomposition temperatures defined by a fixed total mass loss percentage could not directly reflect the intrinsic thermal degradation behavior of the polymer matrix. To address this, the TG curves were further normalized based on the polymer matrix. The onset decomposition temperature (T_5%_) and the maximum weight loss rate temperature in DTG (T_max_) were used for evaluation. The results showed that the main degradation stage of all films was located at 200–300 °C, with similar DTG peak temperatures. This indicated that ONCC@ZnONP did not significantly alter the intrinsic thermal degradation pathway of the OSDF/SA matrix. The apparent half-decomposition temperature based on total mass loss (T_50%_) of the O/CZ8 film was higher than that of the OSDF/SA/ONCC film. This was mainly attributed to the higher inorganic residual mass of ONCC@ZnONP. Meanwhile, the interfacial non-covalent interactions between ONCC@ZnONP and the matrix contributed to a higher char yield and improved apparent thermal stability [51]. This trend was consistent with the filler dispersion observed by SEM and the changes in mechanical properties. In the physically doped ZnO/phycocyanin sago starch system, the maximum thermal degradation temperature (400 °C) was lower than that of pure starch (412 °C) [52]. In contrast, ONCC@ZnONP effectively delayed the thermal cracking of the main chain through a dense interface. It achieved a higher apparent T_50%_ while increasing the char yield, demonstrating better thermal stability.

### 3.6. DSC Analysis of O/CZ Films

Figure 4D showed the thermal transition behavior of the O/CZ films. All OSDF/SA-based samples exhibited a broad endothermic peak at 115–140 °C. ONCC@ZnONP showed no obvious thermal events in this region. This broad peak possibly overlapped with residual moisture evaporation, glycerol thermal events, and polymer melting. Therefore, its peak temperature was denoted as the apparent melting temperature (T_m_). The apparent glass transition temperature (T_g_) was determined by the equal-area method according to ISO 11357-2:2020 [40]. T_m_ first increased and then decreased with increasing ONCC@ZnONP content (O/CZ2: 125.71 °C; O/CZ8: 133.62 °C; O/CZ10: 125.31 °C; OSDF/SA/ONCC: 127.74 °C). T_g_ showed a similar trend (O/CZ2: 76.02 °C; O/CZ8: 97.94 °C; O/CZ10: 72.89 °C; OSDF/SA/ONCC: 78.73 °C). This was due to the formation of interfacial non-covalent interactions between the appropriate amount of ONCC@ZnONP and OSDF chains, which restricted segmental motion. The decrease at 10 wt% was consistent with filler agglomeration, weakened polymer cohesion, and enhanced chain mobility. This result was consistent with the report by A. Król et al. on nanofiller-induced rigid-brittle transitions [53]. The above thermal transition trends were consistent with the filler dispersion/agglomeration observed by SEM and the changes in mechanical properties. Simple blending strategies easily led to nano-ZnO agglomeration and interfacial weakening, and provided limited restriction on polymer chain motion [54]. In contrast, ONCC@ZnONP significantly restricted OSDF chain activity through dense interfacial bonding and endowed the composite films with a higher apparent T_g_.

### 3.7. Mechanical Properties Analysis of O/CZ Films

The influence of ONCC@ZnONP content on the mechanical properties of the O/CZ films was showed in Figure 4E. The underlying mechanisms were explored based on FTIR, XRD, and DSC analyses. The addition of ONCC@ZnONP nanofillers markedly improved the tensile strength (TS) of the O/CZ films. Specifically, the O/CZ8 film showed a TS of 17.23 MPa. This represented a 34.47% increase compared to that of the OSDF/SA/ONCC film. ONCC@ZnONP was incorporated into the matrix as an integrated organic–inorganic dual-functional unit. Its tight interfacial bonding and uniform dispersion allowed for efficient stress transfer [55]. This enhancement was mainly attributed to the interfacial non-covalent interactions between filler surface hydroxyls and OSDF groups. The nucleating effect of ZnO also contributed to this enhancement. The broadening of the hydroxyl peak in the FTIR spectrum supported this conclusion. When the ONCC@ZnONP loading increased to 10 wt%, a slight decrease in tensile strength occurred. This was likely attributed to the local stress concentration caused by filler agglomeration. However, its strength remained higher than that of the OSDF/SA/ONCC film. This partly verified the matrix-supporting role of OSDF. Agar, carrageenan, and CMC films directly doped with ZnO exhibited significantly decreased tensile strength due to weak interfacial interactions [56]. In contrast, ONCC@ZnONP constructed a dense interfacial network. This achieved efficient stress transfer and a simultaneous increase in tensile strength. Regarding elongation at break (EAB), the addition of ONCC@ZnONP did not have a significant overall effect (*p* > 0.05). The EAB of the O/CZ8 film was 22.78% lower than that of the OSDF/SA/ONCC film. This change was likely related to the restricted mobility of matrix molecular chains caused by the rigid ZnO particles. Notably, the EAB increased from 81.97% (O/CZ2 film) to 90.34% (O/CZ8 film). This indicated that an appropriate amount of ONCC formed flexible crosslinking sites within the matrix. As a result, it maintained or even improved ductility while ensuring enhanced strength. Taken together, the mechanical properties of the O/CZ films varied regularly with increasing filler content. This was consistent with the filler dispersion observed by SEM, the enhanced interfacial non-covalent interactions revealed by FTIR, and the restricted chain mobility disclosed by DSC. These findings collectively supported the structure–property relationship of the O/CZ films from both microstructural and thermal perspectives.

### 3.8. Optical Properties Analysis of O/CZ Films

The UV–Vis absorption spectra of the O/CZ films were showed in Figure 4F. Their macroscopic photographs were presented at a higher resolution in Supplementary Figure S1. Compared with OSDF/SA and OSDF/SA/ONCC films, the O/CZ films showed a pronounced absorption band at 365 nm. This corresponded to the characteristic wide bandgap absorption of ZnO [57]. In the 200–300 nm region, all samples showed strong UV absorption. In the 300–400 nm region, the UV-shielding property of the O/CZ films was further enhanced with the addition of ONCC@ZnONP. This phenomenon might be have been attributed to the intrinsic UV absorption of ZnO and nanoscale photophysical effects [58]. It might also have been related to the dispersion state of the filler within the OSDF matrix. At 660 nm in the visible region, all O/CZ films showed a transmittance (T_660_) above 70%. The exception was the O/CZ10 film, where the transmittance slightly dropped to 69.47%. This observation confirmed the relatively uniform dispersion of ONCC@ZnONP in the matrix. The uniform dispersion did not induce significant light scattering. However, a gradual decline in transmittance was observed as the filler loading increased. This phenomenon was attributed to a combination of factors. These included the refractive index mismatch from the higher nanoparticle concentration and localized optical non-uniformity due to minor filler aggregation. Changes in the matrix chain packing caused by hydrogen bonding with the filler also contributed. These findings underscored the importance of balancing UV-shielding performance with optical transparency. They offered insights for optimizing food preservation packaging materials.

### 3.9. Antibacterial Performance Analysis of O/CZ Films

**Figure 5:**
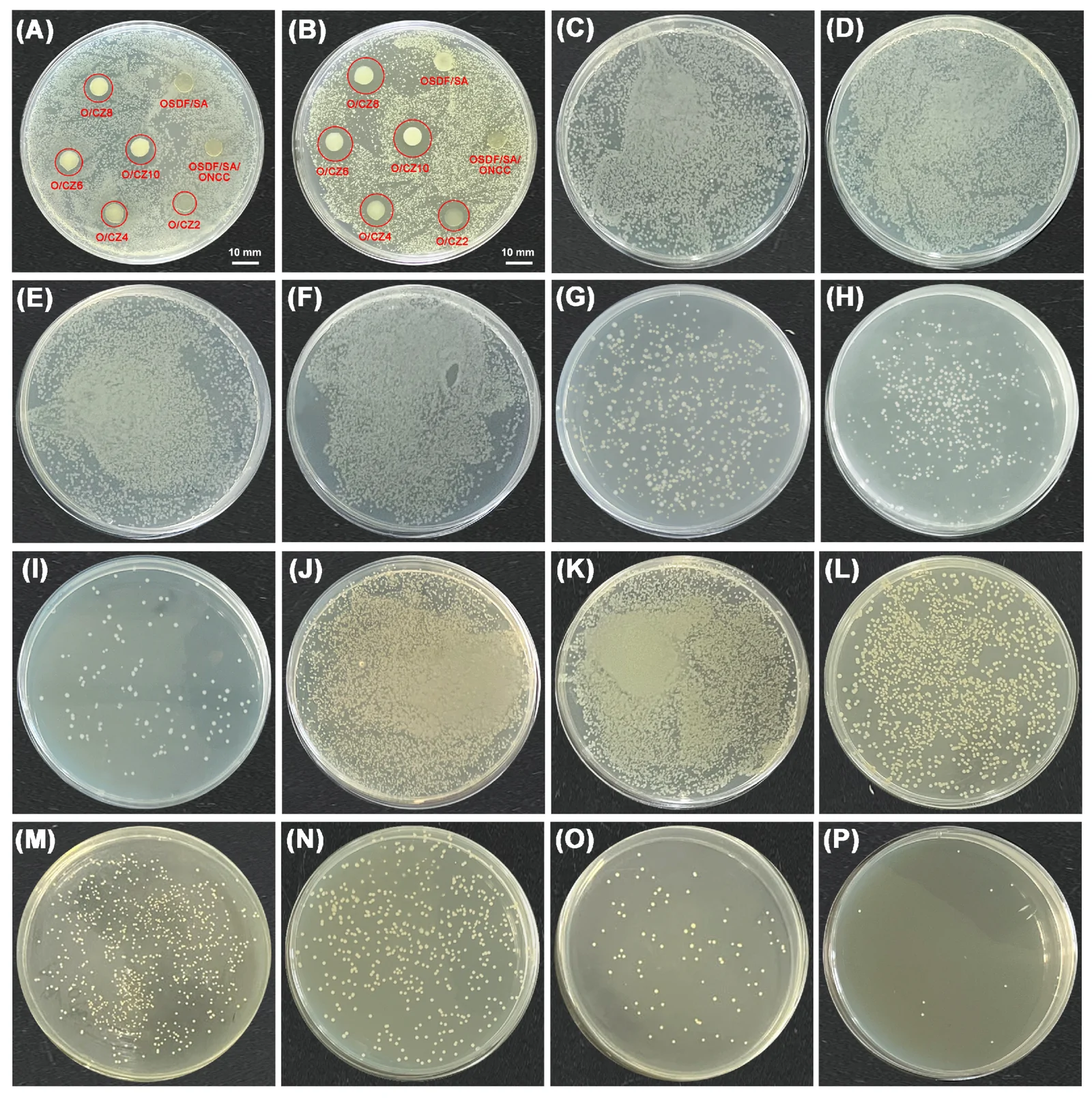
Antibacterial zone of inhibition of O/CZ films against (**A**) *E. coli*, and (**B**) *S. aureus*, antibacterial effect of (**C**) OSDF/SA, (**D**) OSDF/SA/ONCC, (**E**) O/CZ2, (**F**) O/CZ4, (**G**) O/CZ6, (**H**) O/CZ8, and (**I**) O/CZ10 films on *E. coli*, antibacterial effect of (**J**) OSDF/SA, (**K**) OSDF/SA/ONCC, (**L**) O/CZ2, (**M**) O/CZ4, (**N**) O/CZ6, (**O**) O/CZ8, and (**P**) O/CZ10 films on *S. aureus*.

The antibacterial efficacy of the O/CZ films was assessed against *Escherichia coli* (*E. coli*, Gram-negative) and *Staphylococcus aureus* (*S. aureus*, Gram-positive) via inhibition zone and plate count assays. As shown in Figure 5A,B, and Table 1, no inhibition zones were observed for the OSDF/SA or OSDF/SA/ONCC films. This indicated an absence of direct antibacterial activity. However, all O/CZ films incorporating ONCC@ZnONP showed distinct inhibition zones. The inhibition zone diameters (including the 6 mm film disc diameter) enlarged significantly as the filler loading increased (*p* < 0.05). This observation was strongly linked to the antibacterial properties of ZnO within the ONCC@ZnONP hybrid [59,60]. Given that the ONCC content could not be directly compared quantitatively, the OSDF/SA/ONCC film served only as a qualitative baseline for the absence of intrinsic antibacterial activity of pure ONCC.

The photographs of the plate colony counting results for *E. coli* (Figure 5C–I) and *S. aureus* (Figure 5J–P) further confirmed this trend. The OSDF/SA and OSDF/SA/ONCC films showed dense colonies that almost completely covered the agar surface. In contrast, the colony numbers on the O/CZ films incorporating ONCC@ZnONP decreased significantly as the filler loading increased. The O/CZ10 film showed markedly reduced colony numbers for both bacteria. This presented an obvious dose-dependent antibacterial effect [61,62]. Given that the control group had dense colonies exceeding the reliable counting limit (>300 CFU/plate), the inhibition zone diameter was used as the quantitative evaluation index in this study. The plate photographs served only as qualitative evidence. The in situ loaded ZnO/cellulose nanocomposite exhibited enhanced antibacterial activity due to improved particle dispersion [63]. Based on the same in situ growth mechanism, the O/CZ10 film also exhibited prominent inhibition zones against E. coli and S. aureus.

Based on existing research, the antibacterial activity of the O/CZ films was speculated to originate from multiple potential mechanisms of ZnO [64,65]. These mechanisms included the sustained release of Zn^2+^ ions. This release compromised bacterial membrane integrity and disrupted metabolism. Another mechanism involved the generation of reactive oxygen species (ROS) by ZnO in aqueous media. This caused oxidative damage to intracellular substances. Additionally, the penetration of Zn^2+^ through the cell wall disturbed intracellular physiological functions [66]. These potential mechanisms collectively provided a reasonable theoretical basis for the efficient antibacterial performance of the O/CZ films.

### 3.10. Effect of O/CZ Films on Blueberries Preservation

**Figure 6:**
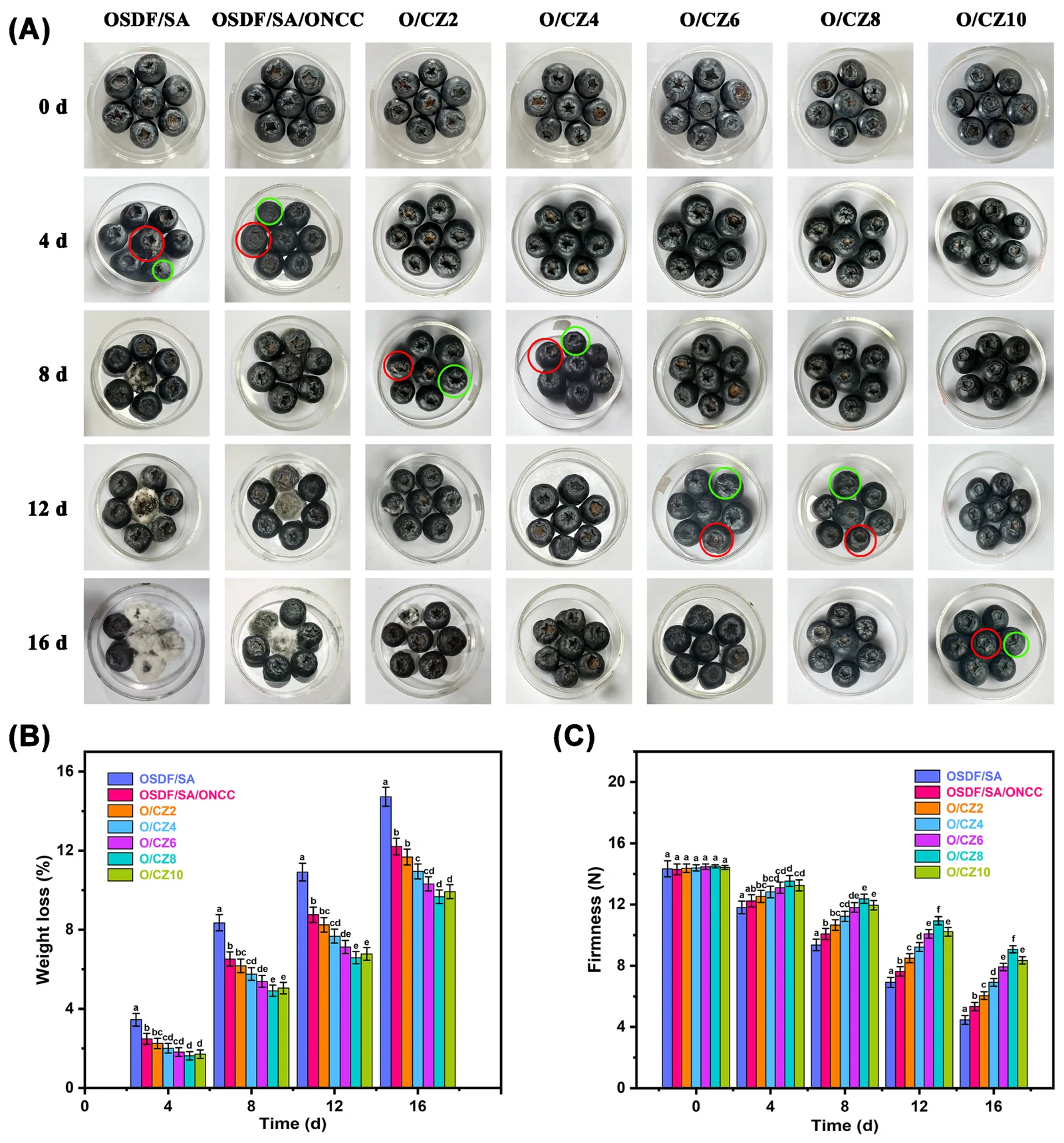
Appearance changes (**A**), weight loss (**B**), and (**C**) firmness of blueberries during storage. Green circles are used to mark the initially wilted blueberries in each group, red circles to mark the initially deteriorated blueberries. Different letters at the same time point indicated significant differences (*p* < 0.05).

Blueberries were prized by consumers for their high nutritional value and distinctive flavor. However, their thin peel and high juice content made them susceptible to water loss and fungal infection during storage. This resulted in fruit softening, surface wrinkling, and spoilage. In the study, the preservation efficacy of the O/CZ films was systematically evaluated using blueberries as the model fruit. Two-way ANOVA revealed that storage time, film treatment, and their interaction had significant effects on the decay rate, weight loss, and firmness of blueberries (*p* < 0.05). Figure 6A showed the visual changes in blueberries from various treatment groups throughout 16 days of storage. After 4 days, mold development was observed on blueberries in the OSDF/SA and OSDF/SA/ONCC groups. By day 16, blueberries in the OSDF/SA group were almost entirely decayed. In contrast, those in the O/CZ10 group showed only slight shriveling and localized spoilage. Consistent with the visual observations, the quantitative decay rate data in Table 2 showed that decay rates in all treatment groups increased with storage time. After 16 days, the OSDF/SA group showed the highest decay rate of 72.35%, significantly higher than the other groups (*p* < 0.05). The decay rates of the O/CZ groups decreased significantly with increasing ONCC@ZnONP loading. Notably, the O/CZ10 group showed a decay rate of only 7.89%. This phenomenon was mainly attributed to the efficient antibacterial activity of ZnO in the O/CZ films (confirmed by the inhibition zone and plate count results). Additionally, the favorable mechanical properties of the O/CZ films helped maintain structural integrity during storage. The UV-shielding property of ZnO might have provided auxiliary antioxidant protection under normal indoor lighting. These factors synergistically delayed fruit decay. These results were consistent with the antibacterial and physical protection effects of composite films reported by Li et al. [67]. Taking a decay rate below 10% as the effective shelf-life endpoint, the chitosan biobased coating maintained blueberry quality for about 12 days at room temperature [68]. In contrast, the O/CZ10 film extended this duration to 16 days. This represented a shelf-life extension of approximately 33%, demonstrating a superior long-term preservation efficacy.

Weight loss rate and firmness served as critical parameters for assessing blueberry freshness and overall quality. Figure 6C showed that the weight loss rates of all treatment groups increased progressively during storage. This phenomenon was mainly attributed to respiratory activity and water transpiration [69]. After 16 days, the OSDF/SA group showed the maximum weight loss rate of 14.72%. Among the O/CZ groups, the weight loss rates declined with rising ONCC@ZnONP content. The O/CZ8 group recorded the lowest value. The O/CZ10 group showed a slight rebound in weight loss rate. This was attributed to a slight decline in barrier performance caused by minor filler agglomeration. This trend showed that an appropriate amount of ONCC@ZnONP enhanced the structural density of the film through interfacial interactions. This possibly helped reduce water evaporation and gas exchange, thereby lowering the weight loss rate. Meanwhile, the UV absorption of ZnO might have partially alleviated photo-oxidative damage [70]. The decrease in blueberry firmness was closely related to cell wall pectin degradation, increased degrading enzyme activity, and moisture loss [71]. As shown in Figure 6C, the firmness of blueberries in all treatment groups decreased during storage. The OSDF/SA group showed the most significant decline, with a firmness of only 4.46 N after 16 days. With increasing ONCC@ZnONP content, the firmness of the O/CZ groups increased significantly. The O/CZ8 group exhibited the highest firmness (9.07 N) after 16 days. In contrast, the O/CZ10 group showed lower firmness than the O/CZ8 group due to higher water loss. The maintenance of firmness was attributed to two factors. First, the antibacterial effect of ZnO reduced the microbial degradation of pectin. Second, the physical coverage of the film helped slow down moisture loss. In summary, the O/CZ10 film exhibited the best performance in inhibiting decay, while the O/CZ8 film showed greater advantages in water retention and anti-softening. Based on the comprehensive balance between antibacterial preservation and physical quality maintenance, the O/CZ8 film achieved the optimal balance between antibacterial activity and structural density through moderate filler dispersion. Therefore, it was identified as the formulation with the best overall preservation performance.

## 4. Conclusions

Novel antibacterial active packaging films (O/CZ films) were successfully developed. Orange peel soluble dietary fiber (OSDF) served as the matrix, and flower-like ONCC@ZnONP acted as a dual-functional reinforcing and antibacterial filler. The results showed that ONCC@ZnONP was uniformly dispersed in the OSDF matrix and formed a dense interface with good interfacial compatibility. Appropriate filler loadings (≤8 wt%) improved the tensile strength through hydrogen bonding and nucleation effects. Additionally, ZnO imparted UV-shielding performance to the films. The O/CZ films showed antibacterial activity against both *Escherichia coli* and *Staphylococcus aureus*. The inhibition zone diameters increasing with filler loading; and the O/CZ10 film showed the largest values (10.43 mm and 15.88 mm, respectively). In the blueberry preservation experiment, the O/CZ10 film showed the strongest antibacterial activity and the lowest decay rate. Based on the comprehensive balance of antibacterial preservation and physical quality maintenance, the O/CZ8 film showed the best performance in reducing weight loss and maintaining firmness. It showed the optimal overall preservation effect. However, the water vapor permeability and oxygen permeability of the films had not been quantitatively determined. Therefore, the preservation effect could only serve as indirect evidence of barrier properties. The migration and toxicological safety of ZnO, as well as the applicability under different storage environments, also remained to be further verified. Future work would focus on the quantitative characterization of barrier properties, migration and safety assessments, and broader application evaluations.

## Figures and Tables

**Table 1:** Zone-of-inhibition of O/CZ films.

Samples	Zone-of-Inhibition (mm)	
	*E. coli*	*S. aureus*
OSDF/SA	/	/
OSDF/SA/ONCC	/	/
O/CZ2	8.24 ± 0.14 ^e^	11.20 ± 0.17 ^e^
O/CZ4	8.59 ± 0.06 ^d^	12.17 ± 0.09 ^d^
O/CZ6	8.98 ± 0.12 ^c^	13.17 ± 0.11 ^c^
O/CZ8	9.30 ± 0.09 ^b^	13.97 ± 0.24 ^b^
O/CZ10	10.43 ± 0.12 ^a^	15.88 ± 0.13 ^a^

Note: The inhibition zone diameter represented the total diameter, including the 6 mm film disc. Results were expressed as mean ± standard deviation (*n* = 3). Values in the same column with different letters were significantly different (*p* < 0.05). “/”, no inhibition zone was observed.

**Table 2:** Decay rate of blueberries during storage.

Samples	Decay Rate/(%)				
	Day 0	Day 4	Day 8	Day 12	Day 16
OSDF/SA	0	14.52 ± 2.2 ^a^	29.14 ± 3.3 ^a^	43.67 ± 3.6 ^a^	72.35 ± 4.1 ^a^
OSDF/SA/ONCC	0	12.37 ± 2.0 ^b^	26.43 ± 3.1 ^a^	32.89 ± 3.3 ^b^	46.82 ± 3.7 ^b^
O/CZ2	0	0	15.21 ± 2.1 ^b^	18.35 ± 2.4 ^c^	32.56 ± 3.2 ^c^
O/CZ4	0	0	12.78 ± 1.9 ^b^	15.62 ± 2.3 ^cd^	29.74 ± 3.1 ^c^
O/CZ6	0	0	0	13.47 ± 2.1 ^d^	18.69 ± 2.2 ^d^
O/CZ8	0	0	0	11.23 ± 1.9 ^d^	15.38 ± 2.1 ^d^
O/CZ10	0	0	0	0	7.89 ± 1.6 ^e^

Note: Results were expressed as mean ± standard deviation (*n* = 3). Two-way analysis of variance (Two-way ANOVA) was used to evaluate the effects of film treatment and storage time. Duncan’s multiple range test was performed for post-hoc multiple comparisons. Values in the same column with different letters were significantly different (*p* < 0.05).

## Data Availability

The original contributions presented in this study are included in the article/Supplementary Materials. Further inquiries can be directed to the corresponding author.

## Supplementary Materials

The following supporting information can be downloaded at: https://www.mdpi.com/article/10.3390/foods15213890/s1, Figure S1: Photograph image of (I) OSDF/SA film, (II) OSDF/SA/ONCC film, (III) O/CZ2 film, (IV) O/CZ4 film, (V) O/CZ6 film, (VI) O/CZ8 film, and (VII) O/CZ10 film.
