# Supplementary material for: Active Films Based on Orange Peel Pomace Soluble Dietary Fiber and Sodium Alginate Reinforced by Dual-Functional Cellulose Nanocrystal/Zinc Oxide Hybrids for Antibacterial Food Packaging
Source: Foods. 2026 Oct 30;15(21):3890. doi: 10.3390/foods15213890 (PMC13762494; doi:10.3390/foods15213890)
Supplement: Supplementary file 1 [file foods-15-03890-s001.zip › foods-4598424-supplementary.pdf]

Supporting Information

Active Films Based on Orange Peel Pomace Soluble Dietary Fiber and Sodium Alginate Reinforced by Dual-Functional Cellulose Nanocrystal/Zinc Oxide Hybrids for Antibacterial Food Packaging

Lili Chen [1], Yuqi Hu [1], Yincai Wu [2], * and Xinjia Jiang [3]

1. Supporting Figures

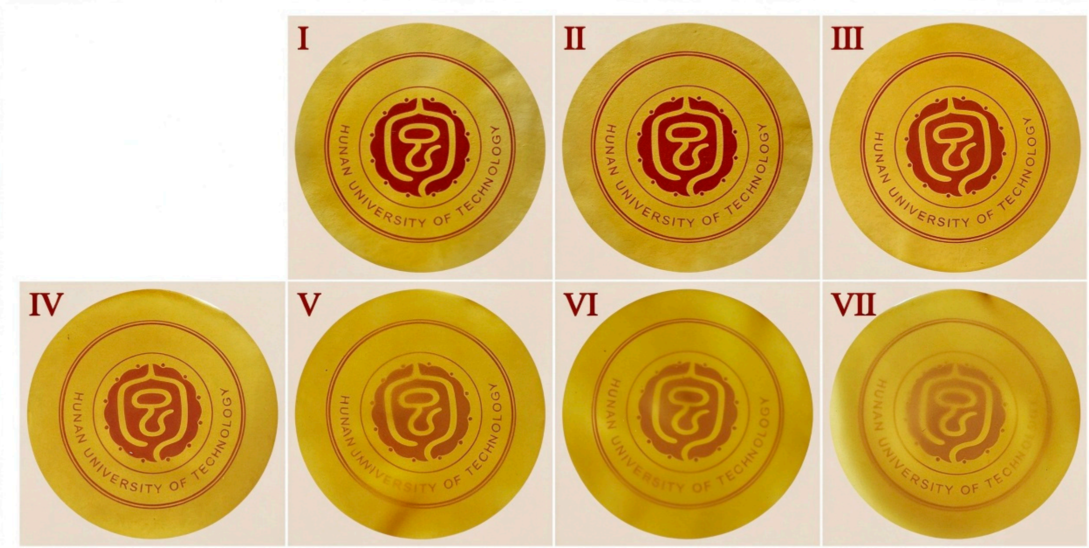

Figure S1. Photograph image of (I) OSDF/SA film, (II) OSDF/SA/ONCC film, (III) O/CZ2 film, (IV) O/CZ4 film, (V) O/CZ6 film, (VI) O/CZ8 film, and (VII) O/CZ10 film.
